# Supplementary material for: Filtering walking actigraphy data in children with unilateral cerebral palsy: A preliminary study
Source: PLoS One. 2024 May 9;19(5):e0303090. doi: 10.1371/journal.pone.0303090 (PMC11081346; doi:10.1371/journal.pone.0303090)
Supplement: S1 Table — (DOCX) [file pone.0303090.s001.docx]

| **S1 Table. Participant Characteristics and Neuroimaging Findings.** | | | | | | | | |
| --- | --- | --- | --- | --- | --- | --- | --- | --- |
| **Child** | **Sex** | **Corrected age (years)** | **Birth term** | **Group allocation** | **Basic pattern of damage** | **Affected hemisphere** | **Cortical lesion** | **Involvement of central nuclei** |
| 1 | M | 5.17 | Full | Experimental | MCA infarction | L | FR, P | BG |
| 2 | F | 5.00 | Pre | Control | MCA infarction | L | FR, P, T | BG, TH |
| 3 | M | 5.42 | Pre | Experimental | N/A | | | |
| 4 | M | 6.50 | Pre | Control | MCA hemorrhagic infarction | L | FR, P | BG, TH |
| 5 | F | 5.00 | Full | Experimental | Dysplastic cortex and brainstem, WMDI | L | FR, P, T | TH |
| 6 | F | 4.42 | Pre | Control | HIE | L > R | T, P, O | CR |
| 7 | F | 9.58 | Full | Experimental | MCA hemorrhagic infarction | L | FR, P, T | BG, TH |
| 8 | M | 4.33 | Pre | Control | Hemorrhage | R | FR, P | TH, BG, PO |
| 9 | M | 4.08 | Full | Control | N/A  No MRI  No MRI | | | |
| 10 | M | 10.58 | Pre | Experimental | N/A  No MRI  No MRI  No MRI | | | |
| 11 | F | 4.00 | Pre | Control | WMDI and hemorrhagic infarction | R | FR, P, T | BG, TH |
| 12 | M | 9.17 | Pre | Experimental | IVH | L | P, T | BG, TH |
| 13 | M | 4.00 | Full | Control | MCA infarction | L | FR, P | TH |
| 14 | M | 4.17 | Full | Experimental | HIE | L | FR, P | BG, TH |
| 15 | F | 4.50 | Full | Control | HIE | L | FR, P, T | TH |
| 16 | F | 5.08 | Full | Experimental | HIE | R | FR | TH, CR |
| 17 | F | 5.83 | Pre | Control | IVH | L | FR, P, O | TH |
| 18 | M | 4.33 | Pre | Experimental | WMDI | R, L | FR | - |
| 19 | M | 6.00 | Full | Control | MCA hemorrhagic infarction | L | FR | BG, TH |
| 20 | M | 4.00 | Full | Experimental | MCA infarction | R | FR, P | BG, TH |
| 21 | F | 5.42 | Pre | Experimental | HIE | R | FR, P, T | BG, TH |
| 22 | F | 4.00 | Full | Experimental | MCA infarction and WMDI | L > R | - | CR |
| M, male; F, female; R, right; L, left; FR, frontal; P, parietal; T, temporal; O, occipital; BG, basal ganglia; WMDI, white matter damage of immaturity; MCA, middle cerebral artery; HIE, hypoxic ischemic encephalopathy; -, no finding; TH, thalamus; CR, corona radiata; PO, pons. | | | | | | | | |
