# Supplementary material for: Filtering walking actigraphy data in children with unilateral cerebral palsy: A preliminary study
Source: PLoS One. 2024 May 9;19(5):e0303090. doi: 10.1371/journal.pone.0303090 (PMC11081346; doi:10.1371/journal.pone.0303090)
Supplement: S2 Table — (DOCX) [file pone.0303090.s002.docx]

| **S2 Table. Column Counts Collected from T0 to T2 (Each Spanning 3 Days) Before and After Filtering.** | | | | |
| --- | --- | --- | --- | --- |
| **Child** | **Total columns** ^a^  **(pre-filtering)** | **Total columns** ^a^  **(post-filtering)** | **Decreased count** | **Reduction rate (%)** |
| 1 | 32,656 | 27,960 | 4,696 | 14.38 |
| 2 | 35,390 | 33,202 | 2,188 | 6.18 |
| 3 | 37,257 | 33,219 | 4,038 | 10.84 |
| 4 | 37,457 | 33,189 | 4,268 | 11.39 |
| 5 | 36,693 | 35,153 | 1,540 | 4.19 |
| 6 | 32,503 | 29,279 | 3,224 | 9.92 |
| 7 | 38,384 | 35,839 | 2,545 | 6.63 |
| 8 | 34,509 | 31,854 | 2,655 | 7.69 |
| 9 | 34,740 | 31,131 | 3,609 | 10.39 |
| 10 | 34,142 | 30,755 | 3,387 | 9.92 |
| 11 | 32,732 | 29,915 | 2,817 | 8.61 |
| 12 | 40,534 | 39,037 | 1,497 | 3.69 |
| 13 | 34,578 | 31,427 | 3,151 | 9.11 |
| 14 | 37,575 | 33,634 | 3,941 | 10.49 |
| 15 | 37,404 | 35,477 | 1,927 | 5.15 |
| 16 | 41,604 | 37,117 | 4,487 | 10.78 |
| 17 | 34,368 | 33,313 | 1,055 | 3.07 |
| 18 | 33,108 | 30,405 | 2,703 | 8.16 |
| 19 | 41,334 | 38,291 | 3,043 | 7.36 |
| 20 | 35,487 | 31,829 | 3,658 | 10.31 |
| 21 | 40,004 | 37,250 | 2,754 | 6.88 |
| 22 | 38,135 | 35,648 | 2,487 | 6.52 |
| **Total** | **800,594** | **734,924** | **65,670** | **8.20** |
| ^a^Data collected over a 10-s epoch.  T0: baseline, T1: post-constraint-induced movement therapy; T2: post-hand-arm bimanual intensive training (experimental group) or equivalent time duration (control group). | | | | |
