# Supplementary material for: Filtering walking actigraphy data in children with unilateral cerebral palsy: A preliminary study
Source: PLoS One. 2024 May 9;19(5):e0303090. doi: 10.1371/journal.pone.0303090 (PMC11081346; doi:10.1371/journal.pone.0303090)
Supplement: S1 Fig — (DOC) [file pone.0303090.s003.doc]

**S1 Fig**

**Enrollment**

**Baseline Measurement (T0)**

**Analysis**

**Post-Intervention Measurement (T1)**

**2-month Follow-up Measurement (T2)**

**Additional In-Lab Measurement**

Assessed for eligibility (n=47)

Excluded (n=24)

  Did not meet the inclusion criteria (n=2)

  Declined to participate because of personal reasons (n=19)

  Did not answer the phone (n=3)

Constraint-induced movement therapy (n=22)

Allocated to intervention (n=22)

Received allocated intervention (n=22)

Analyzed (n=22)

Excluded from analysis (n=0)

Consented/assessed (n=23)

Excluded from analysis (n=1)

 COVID-19 issue (n=1)

Assessed (n=22)

Lost to follow-up (n=0)

Discontinued intervention (n=0)

Assessed (n=22)

Lost to follow-up (n=0)

Assessed (n=4)

Excluded from analysis (n=18)

  Declined to participate because of personal reasons (n=18)
