# Supplementary figures and images for: Filtering walking actigraphy data in children with unilateral cerebral palsy: A preliminary study
Source: PLoS One. 2024 May 9;19(5):e0303090. doi: 10.1371/journal.pone.0303090 (PMC11081346; doi:10.1371/journal.pone.0303090)

**S2 Fig**

**
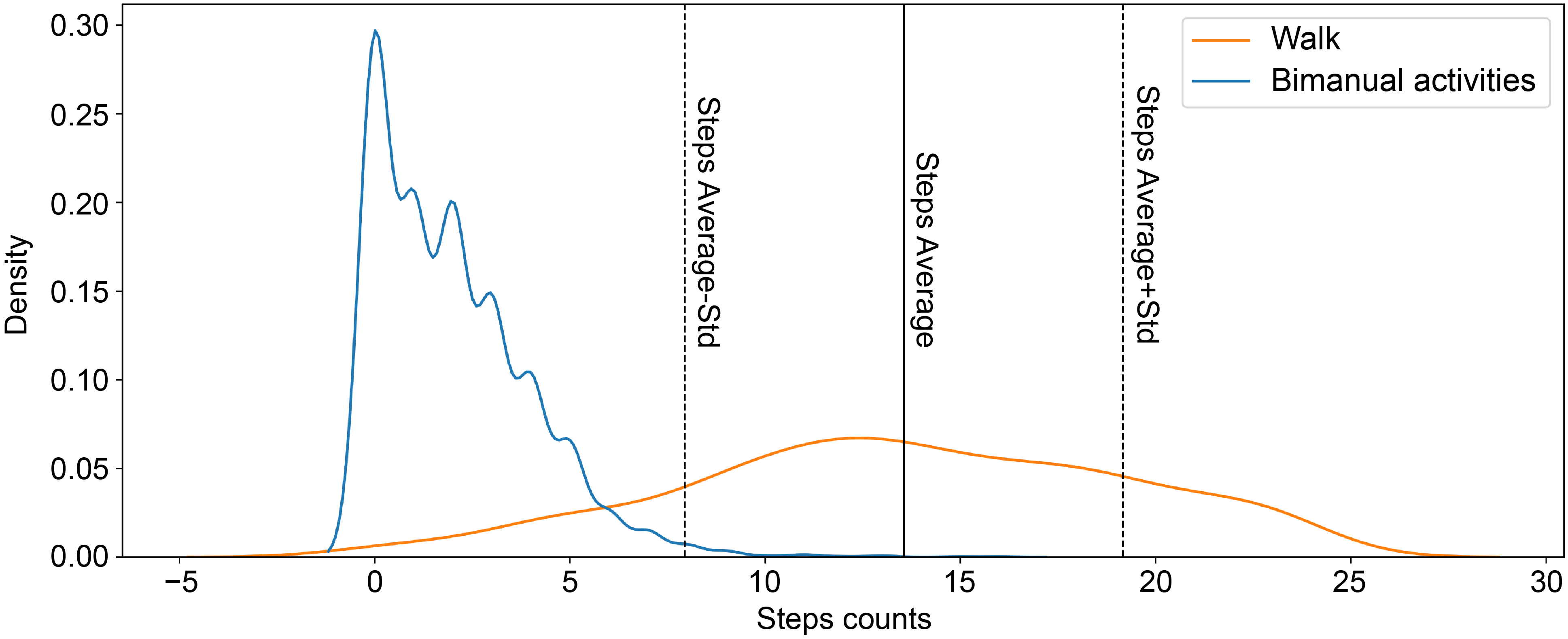
**

Supplement: S2 Fig — The mean step count is represented by a solid black line, and the standard deviation range is shown using dashed black lines. Walking is represented in blue and bimanual tasks are shown in orange. Std, standard deviation. (DOC) [file pone.0303090.s004.doc]
