# Supplementary material for: Filtering walking actigraphy data in children with unilateral cerebral palsy: A preliminary study
Source: PLoS One. 2024 May 9;19(5):e0303090. doi: 10.1371/journal.pone.0303090 (PMC11081346; doi:10.1371/journal.pone.0303090)
Supplement: Attachment — Submitted filename: Point-by-point_response_to_reviewers_PLOSONE.docx [file pone.0303090.s005.docx]

**Point-by-point Response to Reviewer’s Comments**

**Reviewer 1**

**Comment 1: The methods section needs to be rewritten for better readability. Multiple parts are repeated and, hence, redundant. e.g., Page 5 Line 98: "Acceleration data were downloaded and converted into 10-s epochs using ActiLife 6 software (ActiGraph, Pensacola, FL)" and Page 6 Line 116: "After collection, the acceleration data were downloaded and converted into 10-s epochs using the ActiLife 6 software (ActiGraph, Pensacola, FL)."**

Response 1: Thank you for your insightful comment and constructive feedback. We appreciate the opportunity to improve the clarity and readability of our presentation.

In response to your observations, we have carefully revised the entire Methods section to eliminate redundancy. This included addressing the repeated mention of the downloading and conversion of acceleration data, the ActiLife 6 software (ActiGraph, Pensacola, FL), the model name, and, importantly, the duration for which the study participants wore the ActiGraph accelerometers. These repetitions have been removed to ensure a more streamlined and focused narrative.

Furthermore, we have restructured the subsection titles to better reflect the content. The "Participants" subsection has been renamed to "Study Design and Participants" to highlight the inclusion of study design information within this section.

Additionally, we have relocated specific details to sections where they are more contextually appropriate. The explanation of the choice of the ActiGraph wGT3X-BT model, initially placed under "Data Collection," has been moved to "Physical Activity Monitoring Using Accelerometer-Based Monitors." This relocation better situates the rationale for selecting this device within the context of its usage for physical activity monitoring.

Moreover, the detailed description of the bimanual tasks was moved from "Activity Classification and Exclusion in the Test Dataset" to "Data Collection." This adjustment ensures that information on the selection and purpose of these tasks is introduced at a more relevant point in the Methods section, enhancing coherence and the logical flow of information.

Revised manuscript details are provided below.

**Revised Manuscript:**

**Study design and participants**

Children aged 4–12 years diagnosed with UCP due to lesions in the central nervous system participated in this nested study, which was drawn from a two-phase randomized controlled trial (RCT) conducted from July 4th, 2021 to December 22nd, 2022. Data were collected from a tertiary hospital in Seoul, Republic of Korea. The exclusion criteria included severe cognitive dysfunction that hindered the ability to perform simple tasks, untreated seizures, visual or auditory issues that could impede treatment, and a history of musculoskeletal disorders. The hospital review board approved the study (approval no. SMC 2021-04-042), and informed consent was acquired from the parents or legal guardians of the children before enrollment. The study was registered in the Clinical Trials database (clinical trial registration number: NCT04904796).

**Measures**

**Physical activity monitoring using accelerometer-based monitors**

Physical activity information was collected using the ActiGraph wGT3X-BT (ActiGraph, Pensacola, FL), a 4.6×3.3×1.5-cm wireless, tri-axial accelerometer with a dynamic range of ±8 gravitational units at a sampling rate of 30 Hz. The choice of the ActiGraph wGT3X-BT model was underpinned by its documented reliability and validity in previous studies involving ambulatory individuals with CP [10,12]. Vector magnitude average counts (VMA) and VMA ratios were used as actigraphy variables. The VMA was calculated by adding the activity counts in all three axes, while the VMA ratio was determined by computing the natural logarithm (ln) of the ratio between the affected side VMA and less-affected side VMA (ln [affected side VMA/less-affected side VMA]). Acceleration data were downloaded and converted into 10-s epochs using ActiLife 6 software (ActiGraph, Pensacola, FL) and subsequently into activity counts.

**Procedures**

**Data collection**

The primary data collection encompassed children with UCP, selected from the larger cohort of the main RCT. The participants wore accelerometers on their wrists for continuous physical activity monitoring over three consecutive days—specifically Friday, Saturday, and Sunday—to ensure uniformity across all measurement phases: Baseline (T0), post-constraint-induced movement therapy (CIMT) (T1), and a 2-month follow-up (T2). Each day involved more than 10 h of monitoring using accelerometers affixed to both wrists. In parallel, supplementary data collection was undertaken in an in-laboratory setting with a small group of children with UCP. These children engaged in specified activities, encompassing walking and tasks necessitating the coordinated use of both hands. They were likewise outfitted with accelerometers on their wrists. Each in-laboratory session was delineated into a 10-min walking segment followed by a 30-min bimanual task performance segment. Bimanual tasks were carefully selected to represent everyday activities for children with UCP, including sitting and writing at a desk, using a tablet PC, playing board games, turning the pages of a book, and drawing. These activities, necessitating coordinated use of both hands, were distinctively contrasted against the more physically active task of walking.

**Activity classification and filtration in the test dataset**

In this study, 'step count' refers to the number of steps recorded by accelerometers. This metric was chosen as a key variable for its proven effectiveness in previous studies involving children with CP, where distinct activity patterns, including walking, were reliably identified by differing step count values [10,12].

To classify and exclude walking-related data from the dataset, we employed a model developed from step count information gathered during a detailed in-laboratory study with a select group of children with UCP. The reference model was established by analyzing the step count distributions, obtained during in-laboratory sessions that included walking and bimanual task sessions. For the procedure, the step count data underwent a 1-min rolling average. The subsequent classification was achieved by comparing the processed data to established distributions, categorizing each datum as “walking” or “tasks involving both hands.” A computational function was developed to ascertain the probability of the dataset aligning with either “walking” or “tasks involving both hands.” Data from both limbs is needed to indicate walking for a segment to be designated as such. Subsequently, data segments from the main dataset identified as “walking” were systematically filtered, a process we herein refer to as step reduction.

**Comment 2: It is mentioned in the Data Collection section that the subjects were monitored for 3 days (need to mentioned clearly that this occurred at home/in subject's free living environment). There is no mention of what kind of days (weekdays/weekends) the data collection occurred, and whether the days were consistent between T0, T1, T2. Activities at home can change between weekdays and weekends.**

Response 2: Thank you for pointing this out. We have specified in the manuscript that the data collection spanned three consecutive days—Friday, Saturday, and Sunday—for each participant. We intentionally made this selection to capture a comprehensive range of activities typically undertaken by children during weekends, when they are likely not attending school. This scheduling was consistent across all measurement phases: Baseline (T0), post-constraint-induced movement therapy (CIMT) (T1), and a 2-month follow-up (T2), to maintain uniformity in data collection and to allow for a more accurate comparison of physical activity patterns over time.

Revised manuscript details are provided below.

**Revised Manuscript:**

**Procedures**

**Data collection**

The primary data collection encompassed children with UCP, selected from the larger cohort of the main RCT. The participants wore accelerometers on their wrists for continuous physical activity monitoring over three consecutive days—specifically Friday, Saturday, and Sunday—to ensure uniformity across all measurement phases: Baseline (T0), post-constraint-induced movement therapy (CIMT) (T1), and a 2-month follow-up (T2). Each day involved more than 10 h of monitoring using accelerometers affixed to both wrists. In parallel, supplementary data collection was undertaken in an in-laboratory setting with a small group of children with UCP. These children engaged in specified activities, encompassing walking and tasks necessitating the coordinated use of both hands. They were likewise outfitted with accelerometers on their wrists. Each in-laboratory session was delineated into a 10-min walking segment followed by a 30-min bimanual task performance segment.

**Comment 3: The subject count is mentioned multiple times before the demographics are stated. Please describe the patient recruitment, demographics, and exclusions either in the methods section or the results section.**

Response 3: Thank you for your constructive feedback. We have thoroughly reviewed our manuscript with a particular focus on addressing the concern over numerical redundancy within the Procedures section. In response, we have revised our approach to describing the data collection and activity classification processes, ensuring that direct mentions of specific numerical counts of participants involved in these stages are strategically avoided. This adjustment has been made to streamline the narrative and enhance readability, particularly by generalizing descriptions where specific numbers previously appeared, thus aligning with your suggestions for improving clarity in our presentation.

Revised manuscript details are provided below.

**Revised Manuscript:**

**Procedures**

**Data collection**

The primary data collection encompassed children with UCP, selected from the larger cohort of the main RCT. The participants wore accelerometers on their wrists for continuous physical activity monitoring over three consecutive days—specifically Friday, Saturday, and Sunday—to ensure uniformity across all measurement phases: Baseline (T0), post-constraint-induced movement therapy (CIMT) (T1), and a 2-month follow-up (T2). Each day involved more than 10 h of monitoring using accelerometers affixed to both wrists. In parallel, supplementary data collection was undertaken in an in-laboratory setting with a small group of children with UCP. These children engaged in specified activities, encompassing walking and tasks necessitating the coordinated use of both hands. They were likewise outfitted with accelerometers on their wrists. Each in-laboratory session was delineated into a 10-min walking segment followed by a 30-min bimanual task performance segment. Bimanual tasks were carefully selected to represent everyday activities for children with UCP, including sitting and writing at a desk, using a tablet PC, playing board games, turning the pages of a book, and drawing. These activities, necessitating coordinated use of both hands, were distinctively contrasted against the more physically active task of walking.

**Activity classification and filtration in the test dataset**

In this study, 'step count' refers to the number of steps recorded by accelerometers. This metric was chosen as a key variable for its proven effectiveness in previous studies involving children with CP, where distinct activity patterns, including walking, were reliably identified by differing step count values [10,12].

To classify and exclude walking-related data from the dataset, we employed a model developed from step count information gathered during a detailed in-laboratory study with a select group of children with UCP. The reference model was established by analyzing the step count distributions, obtained during in-laboratory sessions that included walking and bimanual task sessions. For the procedure, the step count data underwent a 1-min rolling average. The subsequent classification was achieved by comparing the processed data to established distributions, categorizing each datum as “walking” or “tasks involving both hands.” A computational function was developed to ascertain the probability of the dataset aligning with either “walking” or “tasks involving both hands.” Data from both limbs is needed to indicate walking for a segment to be designated as such. Subsequently, data segments from the main dataset identified as “walking” were systematically filtered, a process we herein refer to as step reduction.

**Comment 4: Page 9 line 187: what is filtration rate? Did you mean reduction rate?**

In our manuscript, "filtration" is used interchangeably with "step reduction." We have clarified this in the manuscript, as follows: "Subsequently, data segments from the main dataset identified as 'walking' were systematically filtered, a process we herein refer to as step reduction." This choice was to ensure our terminology precisely reflects the process undertaken—systematic removal of walking-related data to isolate upper limb activity.

Revised manuscript details are provided below.

**Revised Manuscript:**

**Activity classification and filtration in the test dataset**

In this study, 'step count' refers to the number of steps recorded by accelerometers. This metric was chosen as a key variable for its proven effectiveness in previous studies involving children with CP, where distinct activity patterns, including walking, were reliably identified by differing step count values [10,12].

To classify and exclude walking-related data from the dataset, we employed a model developed from step count information gathered during a detailed in-laboratory study with a select group of children with UCP. The reference model was established by analyzing the step count distributions, obtained during in-laboratory sessions that included walking and bimanual task sessions. For the procedure, the step count data underwent a 1-min rolling average. The subsequent classification was achieved by comparing the processed data to established distributions, categorizing each datum as “walking” or “tasks involving both hands.” A computational function was developed to ascertain the probability of the dataset aligning with either “walking” or “tasks involving both hands.” Data from both limbs is needed to indicate walking for a segment to be designated as such. Subsequently, data segments from the main dataset identified as “walking” were systematically filtered, a process we herein refer to as step reduction.

**Reviewer 2**

**Comment 1: The paper is well organized and the English language quality of the entire paper is really good. However, I do recommend updating few references with up-to-date ones (if applicable).**

Response 1: Thank you for your insightful feedback and recommendation. Following your suggestion, we have carefully reviewed our reference list, changing a reference from the ‘80s to a more recent publication.

**Revised Manuscript:**

1. Klingels, Katrijn, et al. "Upper limb impairments and their impact on activity measures in children with unilateral cerebral palsy." Eur J Paediatr Neurol. 2012;16(5):475-484.

**Reviewer 2**

**Comment 2: I do also see that the authors can give more explanations or discussions on the provided tables or graphs and also the same for the supporting document which can include a visual interpretation of the results or better highlighting the most dominant findings of the work.**

Response 2: Thank you for your insightful feedback. We have augmented the descriptions of the figures to ensure a deeper understanding of the visual representations and their implications.

Revised manuscript details are provided below. Should you have any further suggestions or recommendations, we are eager to hear them.

**Revised Manuscript:**

**Fig 1.** **Depiction of step patterns over time for walking and bimanual tasks.** The collective mean step count is demarcated by a continuous black line, with the standard deviation bounded by dashed black lines. Peak and trough step counts during ambulatory segments are signified by red dashed lines. Ambulatory activity is depicted in orange, symbolizing the dynamic nature of walking, while bimanual tasks are shown in blue, reflecting the controlled movements associated with these activities. This color-coded distinction facilitates a clear visual demarcation between the two types of activities, emphasizing the varying step counts associated with each. Std, standard deviation.

S2 Fig focuses on the distribution of step counts during walking and two-handed tasks. This figure contrasts the frequency of specific step counts, offering insights into the variability and central tendencies of each activity type. Solid black lines indicate the average step count for walking and bimanual activities, with their respective variability represented by dashed lines for standard deviations. The color-coded presentation—walking in orange and bimanual tasks in blue—facilitates an intuitive understanding of the density and distribution patterns, highlighting the distinct characteristics of step counts associated with each type of activity.

**Visualization of data filtration across participants’ actigraphy**

Fig 2 provides a detailed visualization of one participant’s data, illustrating the granularity of how specific segments, such as “Task performance” and “Walking,” were classified and filtered out. This visualization effectively demonstrates how walking-related data, depicted in orange, is isolated and excluded from the overall dataset to focus our analysis on non-ambulatory activities.

**Fig 2.** **A comparative visualization of step counts from one participant’s actigraphy data, focusing on the less-affected (top) and affected (bottom) sides.** Segments marked as 'Walking' in orange are identified for removal, streamlining the dataset for a focused analysis on 'Task performance.' This step is crucial in our methodology, as it ensures the precision of our assessment by excluding walking-induced steps that could obscure the evaluation of bimanual task performance.
